# The impact of head and thorax elevation on respiratory mechanics during cardiopulmonary resuscitation: a multi-model study

**DOI:** 10.1016/j.resplu.2026.101464

**Published:** 2026-08-28

**Authors:** Nicolas Segond, Arnaud Lesimple, Lilian Barlet, Laura Polard, Fanny Lidouren, Emmanuel Charbonney, Dominique Savary, François Morin, Alexandre Bellier, Renaud Tissier, Jean-Christophe Richard, Guillaume Debaty

**Affiliations:** a Univ. Grenoble Alpes, Emergency Department and Mobile Intensive Care Unit, CNRS, UMR 5525, VetAgro Sup, Grenoble INP, TIMC, 38000 Grenoble, France; b Department of Emergency Medicine and Hennepin Healthcare Institute, University of Minnesota, Minneapolis, MN, USA; c Vent’Lab, Medical Intensive Care Unit, Angers University Hospital, Angers, France; d Med2Lab Laboratory, Air Liquide Medical Systems, Antony, France; e Ecole Nationale Vétérinaire d’Alfort, IMRB, AfterROSC Network, F-94700 Maisons-Alfort, France; f Univ Paris Est Créteil, INSERM, IMRB, F-94010 Créteil, France; g Intensive Care Unit, Centre Hospitalier de l’Université de Montéal, Montreal, Canada; h Univ. Grenoble Alpes, Department of Anatomy (LADAF), Grenoble, France

**Keywords:** Cardiac arrest, Ventilation, Head and thorax elevation, Physiology

## Abstract

**Background:**

The impact on ventilation of head and thorax elevation (HTE) and the use of an impedance threshold device (ITD) during cardiopulmonary resuscitation (CPR) remains poorly understood. The aim was to describe the impact on ventilation and absolute lung volumes of HTE vs. horizontal (FLAT) positions, with or without ITD use, across multiple cardiac arrest experimental models.

**Methods:**

This was a cross-over experimental trial conducted using three models: pigs, Thiel-embalmed cadavers, and thawed fresh-frozen cadavers. Two protocols were applied in a 1:1 randomized order: FLAT and HTE positions first, with or without ITD. Primary outcome was the calculation of absolute loss of lung volume (Vloss) below the functional residual capacity. Airway opening pressure (AOP) and respiratory system compliance (Crs) were also calculated, both assessed before CPR initiation. Linear mixed models were used for statistical analysis.

**Results:**

Eight pigs and eight cadavers (4 Thiel, 4 fresh-frozen) were included. In the linear mixed model, there was a significant difference for Vloss for FLAT vs. HTE (*p* = 0.05) with an estimate of 43.9 mL (95%CI: 1.41; 86.4), while no significant difference was observed comparing ITD vs. no-ITD (*p* = 0.28) with an estimate of 23.6 mL (95%CI: −18.9; 66.1). In cadavers, AOP was not significantly different: HTE: 5.9 (2.9–10.3) vs. FLAT: 8.1 (6.4–11.5) cmH_2_O (*p* = 0.07). Crs was not significantly different: HTE: 30.9 (21.3–34.4) vs. FLAT: 26.2 (24.0–30.3) mL/cmH_2_O (*p* = 0.2).

**Conclusions:**

In this multi-model study, HTE during CPR limits loss of lung volume below functional residual capacity.

## Introduction

Despite efforts made to improve the prognosis of out-of-hospital cardiac arrest patients, mortality remains extremely high^1^ calling for new interventions to improve outcomes. The association of the impedance threshold device (ITD) and the active chest decompression (ACD) demonstrated to improve key hemodynamic parameters during cardiopulmonary resuscitation (CPR) in multiple experimental models^2^, ^3^, ^4^, ^5^, ^6^, ^7^ and an improvement of short term survival in clinical settings.^8^, ^9^, ^10^ More recently, it was proposed to associate head and thorax elevation (HTE) to this CPR bundle to decrease intra-cranial pressure and increase cerebral perfusion pressure.^11^, ^12^, ^13^, ^14^, ^15^, ^16^ Beneficial effects on circulation of the association of these interventions (HTE, ACD, ITD) commonly called automated head-up CPR has been previously reported, suggesting that this bundle approach could improve outcomes.^15^, ^16^, ^17^, ^18^ Although ventilation is an increasingly recognized CPR prognosis factor,^19^ the impact of HTE when associated with ITD on ventilation is not yet completely understood and warrants specific physiological investigations. During CPR, chest compressions decrease lung volume below functional residual capacity.^20^ This reduction favors venous return but may also promote the occlusion of small airways, known as airway closure,^21^, ^22^ which may impair alveolar ventilation. By modifying the thorax position related to the abdomen, HTE may change lungs volumes and intrathoracic airway closure, thus improving ventilation. Interestingly, ITD, by limiting flow entering the thorax during chest decompression, may reduce absolute lung volumes and improve venous return. Recent physiological evaluation methods have been proposed to address lung volumes, mechanics and gas exchanges during CPR in both animal and cadaver models.^23^, ^24^, ^25^

The aim of the present study was to describe the impact on ventilation and absolute lung volumes of HTE vs. horizontal (FLAT) positions, with or without ITD use, across multiple cardiac arrest experimental models.

## Methods

### Study design

This was an experimental randomized cross-over trial using different models, comparing predefined physiological ventilatory parameters between two CPR positions: horizontal (FLAT) and HTE with or without an ITD, in intubated models.

The trial was conducted on experimental pig model in National Veterinary School of Alfort; on Thiel cadavers in the Anatomy Department of Trois-Rivières at UQTR (Université du Québec à Trois-Rivières), Canada; and on thawed fresh-frozen cadavers at the Department of Anatomy (LADAF) at Grenoble Medical School (Grenoble Alps University), France. This study was reported following the ARRIVE guidelines^26^ and the checklist is provided in Appendix H. Dedicated ethics committee approved this research for each model (Appendix A).

### Main and secondary endpoints

The effect of position (HTE versus FLAT) and the effect of ITD on ventilation during CPR were evaluated based on the following endpoints:

i. Main endpoint: absolute loss of lung volume (Vloss) below functional residual capacity during CPR in the three models.
ii. Secondary endpoints: inspiratory tidal volume (VTi), expiratory tidal volume (VTe), maximal inspiratory airway pressure (Pmax) and passive ventilation estimated by continuously monitoring volumes generated during decompression (Vdecomp), in the three models.^25^
iii. In the cadaver model, secondary endpoints also included comparisons of airway opening pressure (AOP) and respiratory system compliance (Crs).
iv. In the pig model, the distension ratio, derived from the analysis of the capnogram’s pattern, was compared between the FLAT and HTE positions.^23^
v. Hemodynamic parameters and blood gas measurements were also collected.

### Experimental protocol for pigs

**Preparation:** pigs were instrumented and prepared as usual and previously published.^27^, ^28^ Detailed preparation is available in Appendix A.

**Fig. 1:**
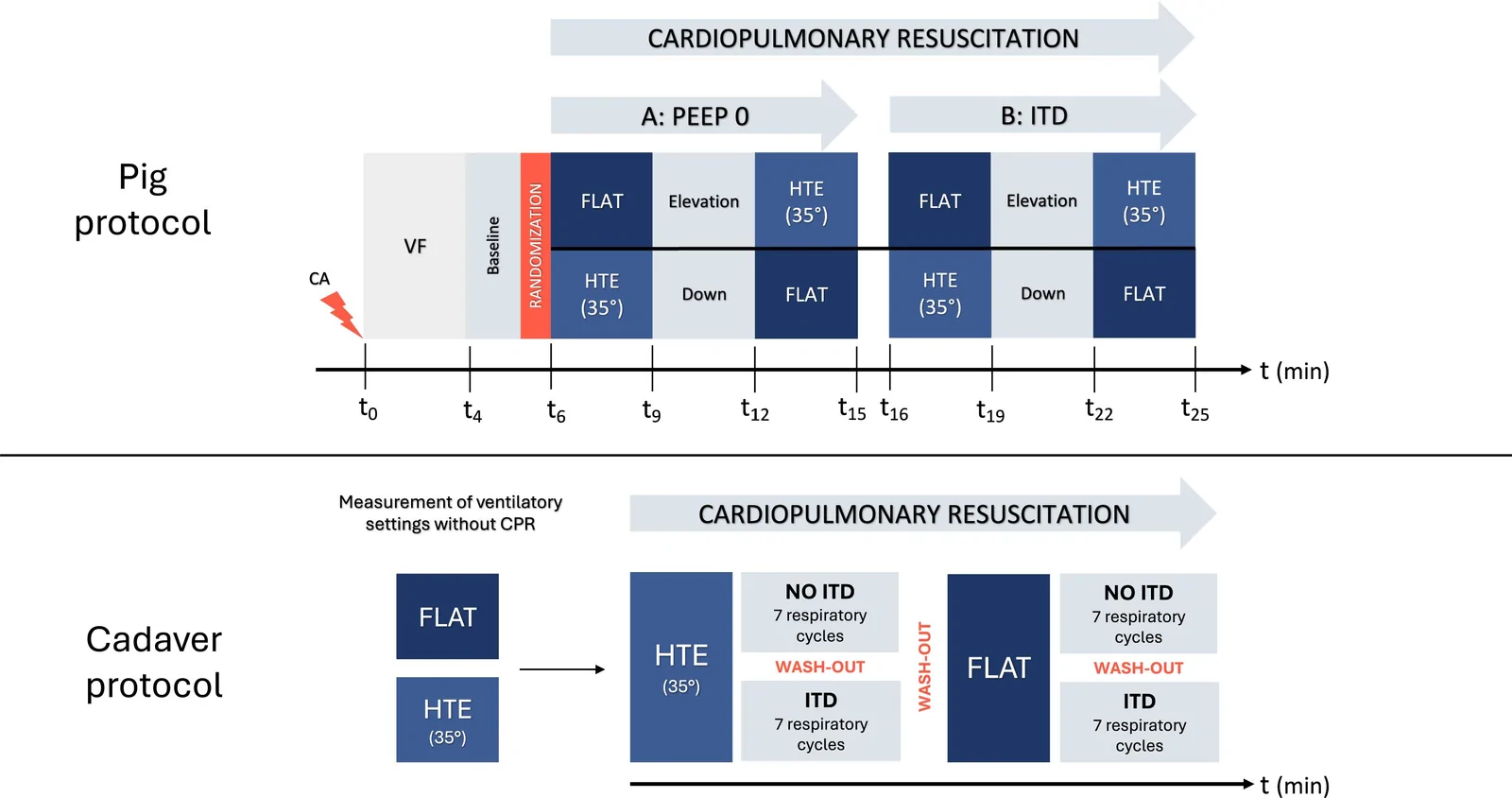
**Study protocol for cadaveric and porcine models**. *PEEP: positive end-expiratory pressure; HTE: head and thorax elevation position; FLAT: flat position; ITD: impedance threshold device; CPR: cardiopulmonary resuscitation. VF: ventricular fibrillation; CA: cardiac arrest.*

**Study protocol (**Fig. 1**):** After instrumentation, ventricular fibrillation was induced by a stimulation probe wire inserted in the right ventricle through the femoral vein catheter. Fibrillation was left untreated during 4 min (no-flow period). Then continuous mechanical chest compressions were started at a rate of 100 per minute using an automated mechanical CPR device with a baseline period of 2 min. During CPR, pigs were ventilated in volume-controlled mode with the following ventilator settings: VTi of 8 mL/kg, inspired oxygen fraction of 100%, ventilator respiratory rate of 10 breaths per minute, positive-end PEEP of 0 cmH_2_O, maximal airway pressure limit of 80 cmH_2_O, inspiratory-expiratory ratio of 1:5, and insufflation time of 1 s. All triggers were turned off.

Then, based on protocol A described in Fig. 1, pigs were randomized in a 1:1 ratio using computer-generated sequence, to start CPR in the HTE position (EleGARD System, Advanced CPR Solutions, United States) or in the horizontal (FLAT) position for 3 min, followed by 2 min of head elevation or head down, and by 3 min of CPR in the FLAT or HTE, depending on the initial randomization group. This sequence was repeated in the protocol B with the use of an impedance threshold device (ITD, Zoll, United States). Protocol A was consistently performed first, followed by Protocol B, without randomization. After 25 min of CPR and the completion of the two protocols, pigs were treated with epinephrine and a shock to attempt a return to spontaneous circulation (ROSC). After ROSC, animals were sacrificed with a lethal dose of pentobarbital (60 mg·kg^−1^).

### Experimental protocol for cadavers

**Preparation:** Detailed cadavers’ preparation is available in Appendix A.

**Study protocol (**Fig. 1**):** after intubation and ventilation start, ventilatory settings measurements were made without chest compression at two positions in the following order with no randomization: FLAT position and HTE position. Mechanical CPR was then started at a rate of 100 compressions per minute. Cadavers were ventilated in volume-controlled mode as previously described for the pig’s protocol. A first sequence at HTE position was started, recording 7 respiratory cycles without ITD and 7 respiratory cycles with ITD. A respiratory cycle was defined as a single inhalation followed by a single exhalation. Each sequence (with and without ITD) was separated with a wash-out period, corresponding to a ventilation without chest compressions. Then, those sequences were repeated in the FLAT position.

### Data processing and measurements

**Fig. 2:**
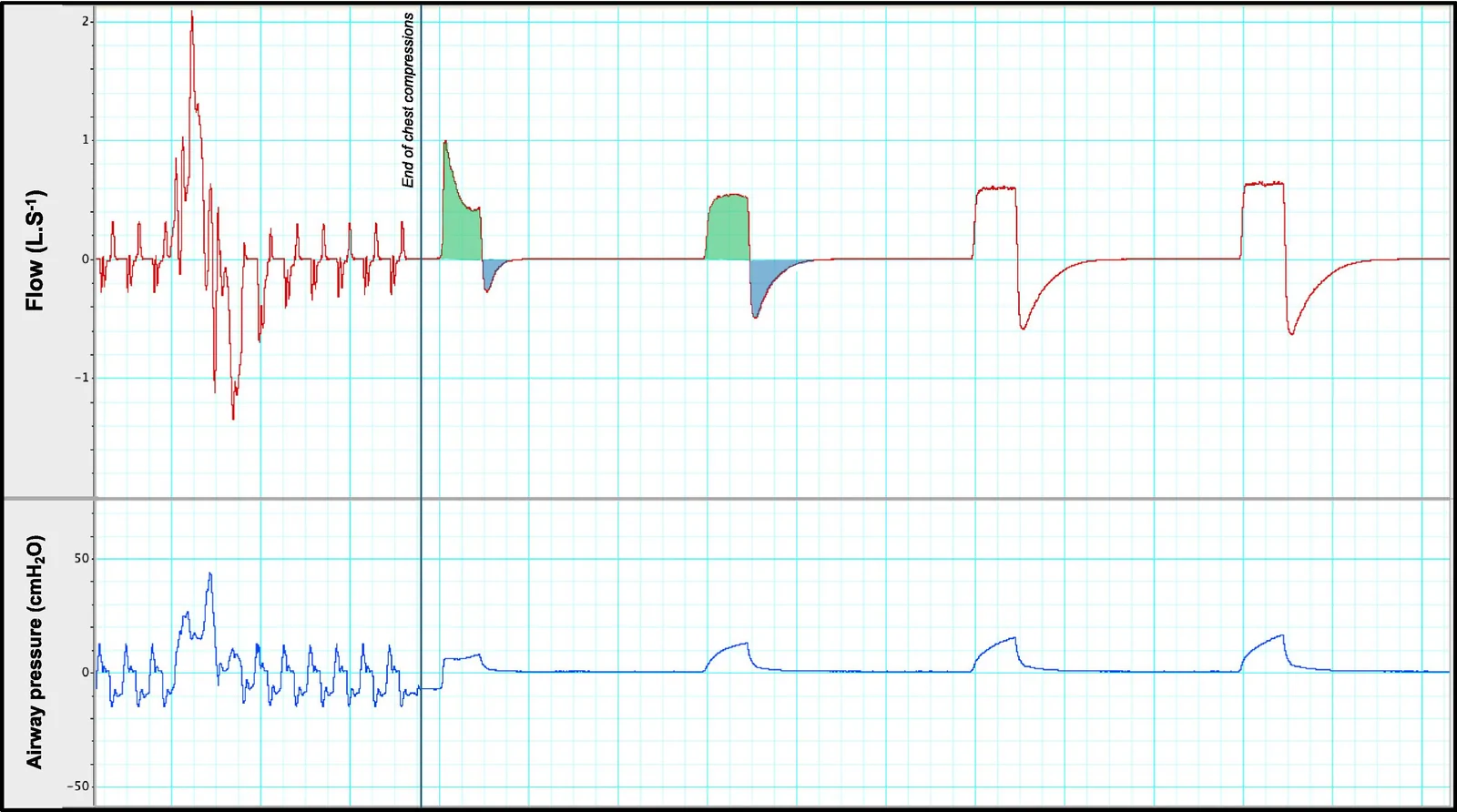
**Example of calculation of absolute loss of lung volume below Functional Residual Capacity (FRC)**. *To estimate the reduction in absolute lung volume below FRC, we integrated the flow signal for two respiratory cycles post-compression. As shown in the figure, this value corresponds to the combined inspiratory (green) and expiratory (blue) volumes immediately following the end of chest compressions.* (For interpretation of the references to color in this figure legend, the reader is referred to the web version of this article.)
ii. **Ventilatory parameters during CPR**: For both animals and cadavers, measurements were derived from flow and pressure signals for each respiratory cycle, and were divided into inspiratory and expiratory phases, using a method described by Segond et al.,^25^ and described in Appendix A.
iii. **Static measurements before CPR**: For cadavers only, static measurements were performed to evaluate presence or absence of airway closure, and measure the AOP in case of airway closure. Airway closure was assessed using the low-flow inflation method (Appendix A). Respiratory system compliance (Crs) was calculated as the ratio between the VTe and the driving pressure, defined as the difference between plateau pressure (Pplat) and total positive end-expiratory pressure (PEEPtot) or AOP (if AOP value was superior to PEEPtot).
iv. **CO_2_ analysis**: For animals only, the distension ratio was measured as previously described by Lesimple et al.^23^ Three ventilatory patterns were reported based on capnogram analysis: intrathoracic airway closure, thoracic distension and regular pattern. Thoracic distension pattern was associated with high tidal volumes and a potential negative impact of ventilation on hemodynamics in the pig model.

All calculations were performed using LabChart Pro software (version 8.1.21, AD Instruments, United States), AcqKnowledge software (version 5.0, Biopac, United States) or Python (Wilmington, United States).

i. **Loss of absolute lung volume**: For both animals and cadavers, loss of absolute lung volume below the functional residual capacity during CPR was estimated by integrating flow signal (volume change) for the two consecutive respiratory cycles following interruption of chest compressions (Fig. 2).

### Statistical analysis

Continuous variables were expressed as mean ± standard deviation (SD) for normally distributed data or as median and first and third quartiles (Q1-Q3) for non-normally distributed data. Categorical variables were presented as counts and percentages. Estimates were reported with their 95% confidence intervals.

For the primary outcome, after normality verification, both experimental models were analyzed using a linear mixed-effects model for loss of lung volume below functional residual capacity, with individual pigs or cadavers as random effect and the thorax position, the use of an ITD and the model type (cadaver or pig) as fixed effects. For the pig protocol, a linear mixed-effects model was applied for each ventilatory measurement, with individual pigs as a random effect and weight, preset tidal volume, thorax position, and use of ITD as fixed effects. For the cadaver protocol, a linear mixed-effects model was also used for each ventilatory parameter measured during ongoing chest compressions, with cadavers as a random effect and height, sex, cadaver model, preset tidal volume, thorax position, and ITD use as fixed effects. Normality was checked for each included variable in the models. Additionally, the normality of the model residuals was assessed visually using a Q-Q plot and a residual histogram, and confirmed statistically using the Kolmogorov-Smirnov test. To compare static measurements prior to CPR, paired *t*-test were performed.

Statistical significance was defined as a two-tailed *p*-value ≤ 0.05. All statistical analyses were performed using Jamovi® software (version 2.3.21.0), with the GAMLj (General Analyses for Linear Models) module.

## Results

### Baseline characteristics

Eight female Yorkshire pigs, weighing 33 ± 5 kg were included. Eight cadavers were included, 4 (50%) with a Thiel preparation and 4 (50%) thawed fresh-frozen. Detailed cadavers and pigs characteristics are shown in Appendices B and C. A total of 384 respiratory cycles for cadavers and 960 respiratory cycles for pigs were analyzed.

### Absolute loss of lung volume below FRC (both animals and cadavers)

**Fig. 3:**
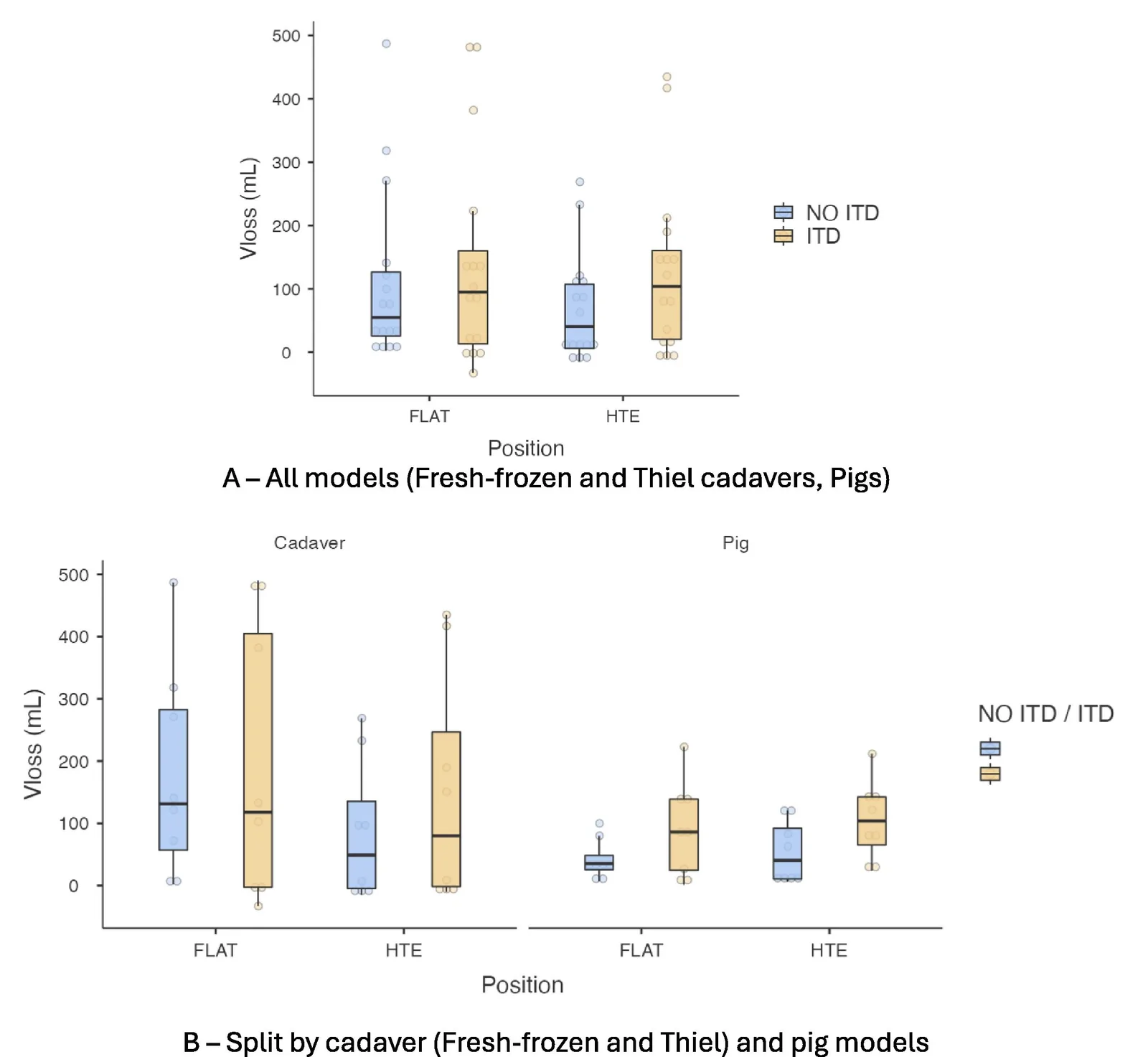
**Box plots representing the absolute loss of lung volume below Functional Residual Capacity (FRC)**. The top panel (A) displays pooled data for the three models (porcine, fresh-frozen and Thielcadavers), while the bottom panels (B) show results for each model independently (Thiel and Fresh-frozen grouped as cadavers model and pig model).

In the three models, without ITD, Vloss was 110.3 ± 136.3 mL in the FLAT position and 61.5 ± 86.2 mL in the HTE position. With ITD, Vloss was 148.7 ± 164.1 mL in the FLAT and 126.8 ± 136.8 in the HTE position (Fig. 3). In the linear mixed model, there was a significant difference for Vloss for FLAT vs. HTE (*p* = 0.05) with an estimate of 43.9 mL (95%CI: 1.41; 86.4), while no significant difference was observed comparing ITD vs. no-ITD (*p* = 0.28) with an estimate of 23.6 mL (95%CI: −18.9; 66.1). In the linear mixed model, the experimental model (pig or cadaver) was a significant factor influencing Vloss (*p* = 0.034) with an estimate of 125 mL (95%CI: 17.5; 233.5).

In the pig model, loss of lung volume without ITD was 42.5 ± 31.7 mL in the FLAT position and 38.8 ± 44.8 mL in the HTE. With ITD, loss of lung volume was 104.6 ± 66.5 mL in the FLAT position and 105.1 ± 62.3 mL in the HTE position.

In the cadaver models, loss of lung volume without ITD was 178.1 ± 187.3 mL in the FLAT position and 84.3 ± 112.9 mL in the HTE. With ITD, loss of lung volume was 192.8 ± 221.0 mL in the FLAT position and 148.5 ± 187.4 mL in the HTE position.

### Ventilatory measurements during CPR (both animals and cadavers)

Results on pigs and cadavers are shown in Table 1.

**Table 1:** Ventilation parameters for each experimental model (pigs and cadavers), stratified by position and the impedance threshold device (ITD) use.

		**PIGS**		**CADAVERS**	
**Parameter**	**Position**	**NO ITD**	**ITD**	**NO ITD**	**ITD**
VTi (mL/kg PBW)	FLAT	6.72 *±* 1.46	6.91 *±* 1.11	8.95 *±* 4.22	7.48 *±* 2.42
	HTE	6.69 *±* 1.70	6.81 *±* 1.12	7.24 *±* 1.72	6.88 *±* 1.90

VTe (mL/kg PBW)	FLAT	7.32 *±* 1.92	6.78 *±* 1.15	5.40 *±* 2.52	6.11 *±* 1.89
	HTE	7.24 *±* 1.84	6.40 *±* 1.36	6.11 *±* 1.82	6.43 *±* 2.04

Pmax inspiratory (cmH_2_O)	FLAT	29.55 *±* 9.62	26.18 *±* 8.30	44.57 *±* 11.90	38.03 *±* 8.41
	HTE	26.89 *±* 8.74	24.33 *±* 7.79	42.68 *±* 11.08	43.15 *±* 13.56

Decompression volume (expiratory reversed airflow) (mL)	FLAT	25.61 *±* 9.63	15.14 *±* 6.39	39.90 ± 48.50	3.19 ± 5.74
	HTE	26.68 *±* 8.77	12.30 *±* 5.36	40.67 ± 49.63	2.64 ± 4.73

Variables are presented as mean ± standard deviation.

For VTi, both ITD and position were significant factors in the linear mixed model in the cadaver model, with a global decrease of VTi in the HTE position (estimates (95%CI); mL/kg: pigs: position: −0.05 (−0.19; 0.08), *p* = 0.45; ITD: 0.17 (0.03; 0.30), *p* = 0.016; cadavers: position: 1.19 (0.67; 1.53), *p* < 0.006; ITD: 0.72 (0.30; 1.13), *p* < 0.001).

For VTe, both ITD and position were significant factors in the linear mixed model for both pigs and cadavers (estimates (95%CI); mL/kg: pigs: position: 0.22 (0.04; 0.39), *p* = 0.01; ITD: 0.69 (0.51; 0.86), *p* < 0.001; cadavers: position: 0.49 (0.06; 0.94), *p* = 0.03; ITD: 0.48 (0.11; 0.84), *p* = 0.01).

For decompression volumes, no difference was observed between FLAT and HTE positions. ITD use was associated with a large decrease of decompression volumes compared to without ITD use. In the linear mixed model, for decompression volumes, ITD use was a significant factor unlike position in the three models (estimates (95%CI); mL: pigs: position: 0.15 (−0.56; 0.85) *p* = 0.68; ITD: 6.13 (5.24; 7.03), *p* < 0.001; cadavers: position: 0.34 (−5.66; 6.34), *p* = 0.85; ITD: 37.15 (23.26; 42.04), *p* < 0.001).

Both ITD and position were significant factors influencing Pmax in the linear mixed model for the two models, with a decrease of Pmax in FLAT position with ITD use and in the HTE position (estimates (95%CI); cmH_2_O: pigs: position: 2.42 (1.67; 3.17), *p* ≤ 0.001; ITD: 2.85 (2.10; 3.61), *p* < 0.001; cadavers: position: 3.20 (1.27; 5.16), *p* = 0.006; ITD: 2.65 (1.06; 4.23), *p* = 0.001).

### Ventilatory parameter measurement prior to CPR (cadavers only)

Median (Q1-Q3) AOP was 5.85 (2.85–10.25) cmH_2_O in the HTE position vs. 8.05 (6.38–11.50) in the FLAT position, with a mean difference of 1.40 (95%CI: −0.18; 2.98); *p* = 0.07. Crs calculation with and without AOP increased with HTE elevation but no significant statistical difference was observed (Table 2). Comparison of static measurements prior to CPR stratified by cadaver model is presented in Appendix E.

**Table 2:** Initial static measurements for each head and thorax position (horizontal (FLAT) position and complete head and thorax elevation (35°)).

**Parameter**	**FLAT position**	**Head and thorax elevation** **(HTE – 35°)**	**Mean difference (95% CI)**	***p***
PEEP total (cmH_2_O)	4.00 (2.50–5.27)	3.70 (1.78–5.20)	0.21 (−0.11 to 0.53)	0.16
Plateau pressure (cmH_2_O)	17.95 (15.03–19.90)	17.10 (14.70–19.02)	0.66 (−0.49 to 1.81)	0.22
Expiratory tidal volume (mL)	343.50 (302.00–378.00)	344.00 (306.75–386.00)	1.00 (−12.8 to 14.8)	0.87
Airway opening pressure (cmH_2_O)	8.05 (6.38–11.50)	5.85 (2.85–10.25)	1.40 (−0.18 to 2.98)	0.07
Respiratory system compliance (mL/cmH_2_O)	26.24 (24.04–30.32)	30.91 (21.25–34.44)	−2.68 (−7.00 to 1.65)	0.20

Variables are presented as median (Q1–Q3). Statistical analysis was performed using paired *t*-test.

Measurements were made before any chest compressions. PEEP: Positive End Expiratory Pressure.

### Distension ratio (animals only)

In the FLAT position, the median distension ratio was 3.51 (2.45–4.19) whereas in the HTE position, distension ratio was 3.43 (2.61–4.12), with no significant statistical difference in the linear mixed model (estimate (95%CI): 3.49 (2.91; 4.06), *p* = 0.09).

### Additional results

In pigs, evolution of hemodynamic parameters is presented in Appendix D. No significant modifications in hemodynamic parameters were observed between groups. Evolution of arterial blood gases during time between each sequence is available in Appendix F.

## Discussion

In this multimodel experimental study, HTE during cardiopulmonary resuscitation compared to horizontal position decreased absolute loss of lung volume below functional residual capacity. The ITD showed no significant impact on the absolute loss of lung volume below functional residual capacity. The present study adds new significant physiological insights regarding the impact of HTE and ITD on ventilation during CPR.

### Impact of head and thorax elevation

The possible effect of HTE on respiratory mechanics has been previously reported and showed to decrease delivered VTi with a decrease of airway pressures in a previous experimental cadaver study.^29^ Moreover, it was demonstrated that HTE could also improve ventilation when using different airway devices.^30^ However, new physiological parameters were assessed in this study to better understand the impact of HTE on ventilation during CPR.

It was previously demonstrated that chest compressions induce a loss of lung volumes below functional residual capacity (FRC).^20^ A dramatic loss of lung volume below the FRC may generate airway closure, which has been shown to impair gas exchange; and may also impede circulation generated by chest compressions through a collapse of veins during decompression. In the present study, HTE was associated with a reduction of Vloss during CPR, suggesting reduced lung collapse and a potential beneficial effect of HTE. This result also suggests the improvement of functional residual capacity in HTE due to a lower position of the diaphragm, as previously observed.^29^

The AOP, corresponding to the minimal airway pressure to open distal airways, related to airway closure,^21^, ^31^, ^32^ was also measured. It was found that the AOP was reduced by elevating head and thorax before CPR in the cadaver model. As airway closure has been shown to limit significantly passive ventilation, this reduction of AOP associated with HTE may be responsible for an increase of passive ventilation. Nevertheless, the reduction of AOP may not have been sufficient to observe this phenomenon. These results are in line with previous studies regarding the impact of position on AOP, resulting potentially to a lower AOP in HTE.^31^, ^33^ The impact seems to be larger in obese patients.^34^ In the present cadaver models, no obese cadaver was included, limiting the effect of HTE on AOP.

### Impact of the impedance threshold valve

The ITD could enhance circulation by preventing the air from entering inside the thorax during the decompression phase, generating negative intrathoracic pressure and promoting venous return.^3^, ^4^, ^5^ Previous studies demonstrated that the ITD decreases passive ventilation but increases VTi.^29^, ^35^ The ITD was associated with a tendency of an increase of absolute loss of lung volume below functional residual capacity and a decrease of passive ventilation. Importantly, active ventilation (Vti) (essential to provide adequate gas exchange) was not influenced by the ITD in the three models. Blood gases and EtCO_2_ were collected in the pigs and are presented in Appendix F, showing the increase of PaCO_2_ and EtCO_2_ without impairment of PaO_2_ with ITD use, as demonstrated in a previous clinical study.^36^ The negative intrathoracic pressure generated by the ITD may improve hemodynamics without impairing active ventilation during CPR.

### Comparison of different experimental models

To date, this study is the first comparing key ventilation parameters during CPR using different experimental models. This method is essential in preclinical research to see if the same trends on different models are observed. Some observations were consistent with all models regarding the absolute loss of lung volume below FRC, the influence of body position and ITD on VTi and decompression volumes. However, the experimental model was a significant factor in the linear mixed model, emphasizing the differences between the models. Indeed, pigs are commonly used to study ventilation strategies,^37^, ^38^ but they have a different lung anatomy with different pulmonary lobes (4 on the right, 2 to the left), a triangle thoracic shape and a different distribution of the interalveolar septa, and the heart is positioned more medially inside the chest, potentially explaining the absence of airway closure in the pig model. Moreover, Thiel and fresh-frozen cadavers showed significant differences in initial static measurements (Appendix E), meaning that the lung physiology of the models was different, leading to a potential attenuation of our results. Despite this, the inclusion of different models remains a strength of this study, improving the translational value of the results. Further experiments are needed to completely understand the impact of these CPR strategies on intra-arrest ventilation.

### Limitations

This study has some limitations. First, although similar, two different protocols were used, one for pig and one for cadavers, but the same data were collected, and same comparisons were made, using the same devices (ventilator, mechanical chest compression device), ensuring translational comparison between the models. Hemodynamic parameters were also evaluated in the porcine models (Appendix D). No differences were observed between the FLAT versus HUP groups or between the ITD versus no-ITD groups. However, these results should be interpreted with caution because of limitations inherent to the study design. Given that the primary focus was on ventilation, the protocol did not incorporate the stepwise elevation of the head and thorax previously described in the literature, a maneuver that may be necessary to optimize and detect the hemodynamic effects of HUP.^16^ HTE was randomized, and CPR was started with HTE before performing CPR in the horizontal position. This initial HTE position has already been shown to be detrimental for hemodynamics since no initial priming was performed.^16^ Active chest compression-decompression, which is a component of the AHUP-CPR bundle of care, was not used in this study. Because active chest compression-decompression could interfere with the physiology of ventilation during resuscitation, further studies are needed to evaluate its specific impact. A time dependent detrimental effect and the limited sample size could have affected the results especially for ITD configurations, which were performed after the sequence with no ITD in the pig model.

## Conclusion

In these animal and cadaver models, head and thorax elevation during CPR limits absolute loss of lung volume below functional residual capacity, suggesting a potential positive effect on ventilation during CPR. The ITD showed no significant impact on the absolute loss of lung volume below functional residual capacity. The interventions appeared to have a more pronounced effect in the cadaveric model than in the porcine model. These inter-model differences in intra-arrest ventilation warrant further investigation.

## CRediT authorship contribution statement

**Nicolas Segond:** Writing – review & editing, Writing – original draft, Visualization, Validation, Methodology, Investigation, Funding acquisition, Formal analysis, Data curation, Conceptualization. **Arnaud Lesimple:** Writing – review & editing, Visualization, Validation, Investigation, Formal analysis, Data curation, Conceptualization. **Lilian Barlet:** Writing – review & editing, Visualization, Validation, Investigation, Data curation. **Laura Polard:** Writing – review & editing, Visualization, Investigation, Data curation. **Fanny Lidouren:** Writing – review & editing, Methodology, Investigation, Data curation. **Emmanuel Charbonney:** Writing – review & editing, Investigation. **Dominique Savary:** Writing – review & editing, Investigation. **François Morin:** Writing – review & editing, Investigation. **Alexandre Bellier:** Writing – review & editing, Methodology. **Renaud Tissier:** Writing – review & editing, Visualization, Validation, Software, Methodology, Investigation, Data curation, Conceptualization. **Jean-Christophe Richard:** Writing – review & editing, Visualization, Validation, Software, Methodology, Investigation, Data curation, Conceptualization. **Guillaume Debaty:** Writing – review & editing, Visualization, Validation, Software, Methodology, Investigation, Data curation, Conceptualization.

## Declaration of competing interest

The authors declare the following financial interests/personal relationships which may be considered as potential competing interests:

AL is working in the Med2Lab funded by Air Liquide Medical Systems.

LP is working in the Med2Lab funded by Air Liquide Medical Systems.

RT reports grants from Air Liquide and grants, shares and personal fees from Orixha, all outside of this work.

JCR reports part time salary for research activities (Med2Lab) from Air Liquide Medical Systems and Vygon and grants from Creative Air Liquide.

GD is a member of the International Liaison Committee on Resuscitation (ILCOR) in the Basic Life Support group.

All other authors declare no competing interests related to this work.

## References

[1] Gräsner J.-T., Herlitz J., Tjelmeland I.B.M., Wnent J., Masterson S., Lilja G. (2021). European Resuscitation Council guidelines 2021: epidemiology of cardiac arrest in Europe. Resuscitation.

[2] Chang M.W., Coffeen P., Lurie K.G., Shultz J., Bache R.J., White C.W. (1994). Active compression-decompression CPR improves vital organ perfusion in a dog model of ventricular fibrillation. Chest.

[3] Lurie K.G., Mulligan K.A., McKnite S., Detloff B., Lindstrom P., Lindner K.H. (1998). Optimizing standard cardiopulmonary resuscitation with an inspiratory impedance threshold valve. Chest.

[4] Lurie K., Voelckel W., Plaisance P., Zielinski T., McKnite S., Kor D. (2000). Use of an inspiratory impedance threshold valve during cardiopulmonary resuscitation: a progress report. Resuscitation.

[5] Plaisance P., Lurie K.G., Vicaut E., Martin D., Gueugniaud P.-Y., Petit J.-L. (2004). Evaluation of an impedance threshold device in patients receiving active compression-decompression cardiopulmonary resuscitation for out of hospital cardiac arrest. Resuscitation.

[6] Plaisance P., Soleil C., Lurie K.G., Vicaut E., Ducros L., Payen D. (2005). Use of an inspiratory impedance threshold device on a facemask and endotracheal tube to reduce intrathoracic pressures during the decompression phase of active compression-decompression cardiopulmonary resuscitation. Crit Care Med.

[7] Metzger A.K., Herman M., McKnite S., Tang W., Yannopoulos D. (2012). Improved cerebral perfusion pressures and 24-h neurological survival in a porcine model of cardiac arrest with active compression-decompression cardiopulmonary resuscitation and augmentation of negative intrathoracic pressure. Crit Care Med.

[8] Aufderheide T.P., Frascone R.J., Wayne M.A., Mahoney B.D., Swor R.A., Domeier R.M. (2011). Standard cardiopulmonary resuscitation versus active compression-decompression cardiopulmonary resuscitation with augmentation of negative intrathoracic pressure for out-of-hospital cardiac arrest: a randomised trial. Lancet Lond Engl.

[9] Plaisance P., Lurie K.G., Vicaut E., Adnet F., Petit J.L., Epain D. (1999). A comparison of standard cardiopulmonary resuscitation and active compression-decompression resuscitation for out-of-hospital cardiac arrest. French Active Compression-Decompression Cardiopulmonary Resuscitation Study Group. N Engl J Med.

[10] Wolcke B.B., Mauer D.K., Schoefmann M.F., Teichmann H., Provo T.A., Lindner K.H. (2003). Comparison of standard cardiopulmonary resuscitation versus the combination of active compression-decompression cardiopulmonary resuscitation and an inspiratory impedance threshold device for out-of-hospital cardiac arrest. Circulation.

[11] Debaty G., Shin S.D., Metzger A., Kim T., Ryu H.H., Rees J. (2015). Tilting for perfusion: head-up position during cardiopulmonary resuscitation improves brain flow in a porcine model of cardiac arrest. Resuscitation.

[12] Moore J.C., Holley J., Segal N., Lick M.C., Labarère J., Frascone R.J. (2018). Consistent head up cardiopulmonary resuscitation haemodynamics are observed across porcine and human cadaver translational models. Resuscitation.

[13] Moore J.C., Salverda B., Rojas-Salvador C., Lick M., Debaty G., Lurie K.G. (2021). Controlled sequential elevation of the head and thorax combined with active compression decompression cardiopulmonary resuscitation and an impedance threshold device improves neurological survival in a porcine model of cardiac arrest. Resuscitation.

[14] Pourzand P., Moore J., Metzger A., Salverda B., Suresh M., Arango S. (2024). Hemodynamics, survival and neurological function with early versus delayed automated head-up CPR in a porcine model of prolonged cardiac arrest. Resuscitation.

[15] Pourzand P., Moore J., Metzger A., Suresh M., Salverda B., Hai H. (2025). Intraventricular pressure and volume during conventional and automated head-up CPR. Resuscitation.

[16] Moore J.C., Salverda B., Lick M., Rojas-Salvador C., Segal N., Debaty G. (2020). Controlled progressive elevation rather than an optimal angle maximizes cerebral perfusion pressure during head up CPR in a swine model of cardiac arrest. Resuscitation.

[17] Moore J.C., Duval S., Lick C., Holley J., Scheppke K.A., Salverda B. (2022). Faster time to automated elevation of the head and thorax during cardiopulmonary resuscitation increases the probability of return of spontaneous circulation. Resuscitation.

[18] Moore J.C., Bartos J.A., Matsuura T.R., Yannopoulos D. (2017). The future is now: neuroprotection during cardiopulmonary resuscitation. Curr Opin Crit Care.

[19] Idris A.H., Aramendi Ecenarro E., Leroux B., Jaureguibeitia X., Yang B.Y., Shaver S. (2023). Bag-valve-mask ventilation and survival from out-of-hospital cardiac arrest: a multicenter study. Circulation.

[20] Cordioli R.L., Lyazidi A., Rey N., Granier J.-M., Savary D., Brochard L. (2016). Impact of ventilation strategies during chest compression. An experimental study with clinical observations. J Appl Physiol.

[21] Heil M., Hazel A.L., Smith J.A. (2008). The mechanics of airway closure. Respir Physiol Neurobiol.

[22] Guérin C., Cour M., Argaud L. (2021). Airway closure and expiratory flow limitation in acute respiratory distress syndrome. Front Physiol.

[23] Lesimple A., Fritz C., Hutin A., Charbonney E., Savary D., Delisle S. (2022). A novel capnogram analysis to guide ventilation during cardiopulmonary resuscitation: clinical and experimental observations. Crit Care Lond Engl.

[24] Duhem H., Terzi N., Segond N., Bellier A., Sanchez C., Louis B. (2023). Effect of automated head-thorax elevation during chest compressions on lung ventilation: a model study. Sci Rep.

[25] Segond N., Wittig J., Kern W.J., Orlob S. (2025). Towards a common terminology of ventilation during cardiopulmonary resuscitation. Resuscitation.

[26] du Sert N.P., Ahluwalia A., Alam S., Avey M.T., Baker M., Browne W.J. (2020). Reporting animal research: explanation and elaboration for the ARRIVE guidelines 2.0. PLoS Biol.

[27] San Geroteo J., Jendoubi A., Lidouren F., Watanabe N., Abi Zeid Daou Y., Hutin A. (2026). Evaluation of systemic and cerebral hemodynamics after systematic and early extracorporeal cardiopulmonary resuscitation in swine. Resusc Plus.

[28] Watanabe N., Bois A., Lidouren F., Abi Zeid Daou Y., Jendoubi A., Gaborieau B. (2025). Total liquid ventilation in a porcine model of severe acute respiratory distress syndrome using a new generation of liquid ventilator. Intensive Care Med Exp.

[29] Segond N., Terzi N., Duhem H., Bellier A., Aygalin M., Fuste L. (2023). Mechanical ventilation during cardiopulmonary resuscitation: influence of positive end-expiratory pressure and head-torso elevation. Resuscitation.

[30] Segond N., Fischer M., Fontecave-Jallon J., Podsiadlo P., Lurie K., Bellier A. (2025). Effect of different airway devices on ventilation during cardiopulmonary resuscitation. Resuscitation.

[31] Garland A., Hopton P. (2022). Airway closure in anaesthesia and intensive care. BJA Educ.

[32] Hedenstierna G. (2020). Complete airway closure. Anesthesiology.

[33] Pavlovsky B., Lesimple A., Richard J.-C., Chean D., Courtais A., Leprovost P. (2025). Airway opening pressure in mechanically ventilated patients: regional distribution and impact of body position. Crit Care Lond Engl.

[34] Bihari S., Wiersema U.F. (2024). Changes in respiratory mechanics with trunk inclination differs between patients with ARDS with and without obesity. Chest.

[35] Morin F., Polard L., Fresnel E., Richard M., Schmit H., Martin-Houitte C. (2024). A new physiological manikin to test and compare ventilation devices during cardiopulmonary resuscitation. Resusc Plus.

[36] Debaty G., Segond N., Duhem H., Crespi C., Behouche A., Boeuf J. (2024). Comparison of end tidal CO_2_ levels between automated head up and conventional cardiopulmonary resuscitation: a pre-post intervention trial. Resuscitation.

[37] Vognsen M., Fabian-Jessing B.K., Secher N., Løfgren B., Dezfulian C., Andersen L.W. (2017). Contemporary animal models of cardiac arrest: a systematic review. Resuscitation.

[38] Li J.-C., Segond N., Lesimple A., Hutin A., Goutchtat R., Drennan I. (2026). Evaluation of ventilatory parameters reporting in large animal models of cardiac arrest: a scoping review. Resusc Plus.

