# Supplementary material for: The impact of head and thorax elevation on respiratory mechanics during cardiopulmonary resuscitation: a multi-model study
Source: Resusc Plus. 2026 Aug 28;31:101464. doi: 10.1016/j.resplu.2026.101464 (PMC13579023; doi:10.1016/j.resplu.2026.101464)
Supplement: Supplementary file 1 [file mmc1.docx]

**The impact of head and thorax elevation on respiratory mechanics during cardiopulmonary resuscitation: a multi-model study.**

**APPENDICES**

**Appendix A – Methods Details**

***Ethical Statement***

The pig model experiment was approved by the ethics committee for animal research Cometh – 016, in compliance with the procedure for the care and sacrifices of study animals of the European Community Standards on the Care and Use of Laboratory Animals.

The thawed fresh frozen cadavers experiment was performed in France in LADAF laboratory, the human bodies used in this study were donated for medical science by the donors. Written and witnessed consent to donate their bodies to science for anatomical and teaching purposes was obtained prior to death. This donation was free, anonymous, and regulated by French legislation. The study was approved by the LADAF ethical committee.

The Thiel cadavers study was performed in the Anatomy Department of UQTR, Canada; cadavers came from a specific donation program and experiments were conducted in accordance with Canadian regulations after approval from the local Research Ethics Board (CER-09-148-06.05).

**Detailed pig’s preparation:**

Pigs weighing 33±5 kg were anesthetized with a mixture of tiletamine (10 mg.kg^-1^ i.v.), zolazepam (10 mg.kg^-1^, i.v.), propofol (10 mg.kg^-1^.h^-1^ i.v.) and methadone (0.3 mg/kg^-1^ i.m.). Then, they were intubated and mechanically ventilated (Monnal T60, Air Liquide, Medical Systems, Antony, France) with the following settings: inspired fraction of oxygen = 30%, tidal volume = 8 ml/kg, positive end expiratory pressure (PEEP) = 5 cmH_2_O and respiratory rate = 25±5. Body temperature was controlled for a core temperature at 38°C. Animals were monitored by a five-lead electrocardiogram. Oxygen saturation (SpO_2_) and CO_2_ using the Monnal system (Irma CO_2_ probe, Masimo Corporation CA, USA) were recorded. Catheters were inserted into femoral vein and artery for the evaluation of aortic and right atrial pressure, respectively. An intracranial pressure probe was inserted after trepanation (Millar®, Houston, USA). After a period of stabilization, animals were paralyzed by rocuronium (1 mg.kg^-1^). The mechanical compression device, a LUCAS^TM^ (Lucas 3, Stryker, MI, USA) was placed in a controlled and secured position. Hemodynamic parameters were continuously recorded throughout the experimental protocol (HEM, Notocord, Croissy-sur-Seine, France). Parameters continuously recorded were airway pressure (cmH2O), aortic blood pressure (mmHg), right atrial pressure (mmHg), intracranial pressure (mmHg), EtCO_2_. Coronary perfusion pressure was calculated as aortic blood pressure minus right atrial pressure and cerebral perfusion pressure was calculated as mean arterial pressure minus intracranial pressure. Airway flow was obtained from the ventilator flow sensor. All parameters were sampled at 200 Hz.

**Detailed cadavers’ preparation:**

Thiel cadavers were prepared using a special formalin-based preparation method [28]. Thiel cadavers were stored in a tank immersed in the liquid preparation. Flow and Paw were measured using a Pneumotachograph (Fleisch n°3, Lausanne, Switzerland) positioned proximally to the endotracheal tube and connected to a Biopac module system (Goleta, CA, USA). Concerning fresh-frozen cadavers, the cadavers were frozen immediately upon arrival at LADAF. They were removed from the freezer, stored at -20°C (-4°F), and thawed 5 to 7 days before use. Flow and pressure signals were sampled at 200 Hz and recorded using an analog-digital system (Powerlab, AD Instruments, New Zealand) and its software (LabChart, version 8.1.28). Before each experiment, all cadavers were intubated and ventilated for 30 minutes.

**Ventilatory parameters calculated during ongoing chest compressions:**

For the inspiratory phase, the inspiratory tidal volume (VTi) was defined as the volume of air entering the lungs during inspiration (expressed in ml and ml/kg). Maximum inspiratory airway pressure (Pmax) was defined as the peak pressure during inspiration. Passive ventilation was defined as the volume induced by chest decompression during the expiratory phase (also called expiratory reversed airflow). The volume induced by chest compressions during the expiratory phase was also calculated (Vdecomp).

**Detailed distension ratio calculation:**

Thoracic distension was quantified by computing the distension ratio based on the analysis of the area under the CO2 curve; a high distension ratio being associated with high thoracic distension. The distension ratio was calculated as the ratio between the initial area under the CO2 curve without oscillation (AUC1) and the area of the consecutive normal CO2 oscillation (AUC2). A distension ratio of 2 (AUC1 is two times superior to AUC2) was arbitrarily defined as a cut-off value; considering that the loss of oscillations in case of thoracic distension includes at least two inefficient chest decompressions (distension ratio ≥ 2).

**Low-flow insufflation method for the calculation of AOP:**

During volume-controlled ventilation, a constant low inspiratory flow of 5 L/min was applied in order to minimize the resistive component of airway pressure. AOP was identified as the airway pressure value associated with a sudden change in slope on the pressure-volume curve (inflection point), indicating the opening of previously collapsed or closed airways. If no inflection point was observed, the cadaver was considered as having no airway closure and thus no AOP.

**Appendix B - Detailed pigs characteristics**

*VT set : tidal volume set on ventilator.*

| Pig | Sex | Weight (kg) | Vt weight predicted (mL) | Vt set (mL) |
| --- | --- | --- | --- | --- |
| 550 | F | 33 | 264 | 260 |
| 551 | F | 34 | 272 | 270 |
| 629 | F | 29 | 232 | 240 |
| 630 | F | 26 | 208 | 210 |
| 698 | F | 32 | 256 | 250 |
| 699 | F | 32 | 256 | 250 |
| 734 | F | 44 | 352 | 330 |
| 735 | F | 35 | 280 | 270 |

**Appendix C - Detailed cadavers characteristics**

*BMI : body mass index ; Vt PBW : net tidal volume adjusted on predicted body weight ; VT set : tidal volume set on ventilator.*

| Type of Cadaver | Cadaver | Sex | Age (years) | Height (meters) | Weight (kg) | BMI (kg/m2) | Post-mortem delay (days) | Vt PBW (mL) | Vt set (mL) |
| --- | --- | --- | --- | --- | --- | --- | --- | --- | --- |
| Thiel | 1 | F | 85 | 1.68 | 50 | 17.7 | 121 | 258 | 260 |
|  | 2 | H | 83 | 1.70 | 68 | 23.5 | 90 | 372 | 390 |
|  | 3 | F | 73 | 1.65 | 61 | 22.4 | 78 | 342 | 340  260 |
|  | 4 | F | 75 | 1.52 | 55 | 23.8 | 77 | 270 | 270  340 |
| Fresh Frozen | 5 | F | 71 | 1.62 | 65 | 24.8 | - | 432 | 430 |
|  | 6 | F | 95 | 1.60 | 55 | 21.5 | - | 416 | 420 |
|  | 7 | H | 80 | 180 | 80 | 24.7 | 32 | 600 | 600 |
|  | 8 | H | 71 | 175 | 65 | 21.2 | 31 | 560 | 560 |
| Means ± std  or n (%) | - | H 3 (37.5%)  F 5 (62.5%) | 79.1 ± 8.2 | 1.67 ± 0.09 | 62.4 ± 9.4 | 22.5 ± 2.1 | 31.5 ± 0.7 | 406.3 ± 112.2 | 408.8 ± 110.7 |

**Appendix D – Hemodynamics parameters in pigs for each sequence**

*CePP: Cerebral Perfusion Pressure ; PIC: intracranial pressure; Ao: aortic blood pressure; RAP: Right Atrial Pressure, CoPP: Coronary Perfusion Pressure*

*FLAT and HTE groups were compared using paired t-test.*

*Variables are presented as mean ± std.*

| Pressure | Position | Baseline | FLAT | HTE | *p* |
| --- | --- | --- | --- | --- | --- |
| Systolic CePP | PEEP 0 | 103.37 ± 21.09 | 57.90 ± 32.57 | 56.93 ± 23.22 | 0.86 |
|  | ITD |  | 41.48 ± 17.33 | 50.41 ± 21.42 | 0.43 |
| Mean CePP | PEEP 0 | 80.58 ± 18.77 | 20.07 ± 6.91 | 23.34 ± 6.65 | 0.42 |
|  | ITD | - | 23.99 ± 6.80 | 18.47 ± 5.54 | 0.15 |
| Diastolic CePP | PEEP 0 | 64.68 ± 19.05 | 12.68 ± 5.40 | 11.84 ± 10.57 | 0.84 |
|  | ITD | - | 16. 24 ± 7.50 | 8.18 ± 7.49 | 0.10 |
| Systolic PIC | PEEP 0 | 13.09 ± 3.52 | 35.81 ± 8.37 | 29.67 ± 18.28 | 0.25 |
|  | ITD | - | 22.25 ± 6.26 | 22.83 ± 13.93 | 0.87 |
| Mean PIC | PEEP 0 | 10.83 ± 3.43 | 18.21 ± 4.58 | 15.42 ± 9.55 | 0.40 |
|  | ITD | - | 11.18 ± 4.27 | 12.10 ± 8.28 | 0.83 |
| Diastolic PIC | PEEP 0 | 9.05 ± 3.46 | 8.90 ± 4.64 | 6.48 ± 6.54 | 0.42 |
|  | ITD | - | 4.04 ± 3.33 | 4.47 ± 6.39 | 0.92 |
| Systolic Ao | PEEP 0 | 111.01 ± 23.18 | 90.39 ± 27.15 | 77.43 ± 20.64 | 0.09 |
|  | ITD | - | 65.60 ± 10.10 | 69.83 ± 20.20 | 0.57 |
| Mean Ao | PEEP 0 | 91.76 ± 20.53 | 42.66 ± 7.93 | 38.52 ± 7.10 | 0.27 |
|  | ITD | - | 34.49 ± 6.02 | 33.38 ± 9.47 | 0.67 |
| Diastolic Ao | PEEP 0 | 75.85 ± 20.17 | 11.38 ± 11.60 | 12.22 ± 12.32 | 0.75 |
|  | ITD | - | 11.48 ± 9.22 | 9.67 ± 13.31 | 0.51 |
| Diastolic RAP | PEEP 0 | 2.02 ± 4.38 | 10.84 ± 3.75 | 11.86 ± 4.21 | 0.23 |
|  | ITD | - | 11.34 ± 4.72 | 13.70 ± 5.84 | 0.27 |
| CoPP | PEEP 0 | 91.69 ± 20.19 | 16.11 ± 8.42 | 12.68 ± 6.24 | 0.16 |
|  | ITD | - | 12.74 ± 6.49 | 7.72 ± 10.72 | 0.14 |

**Appendix E – Initial static measurements stratified by cadaver model.**

*Variables are presented as median (Q1 – Q3).*

*Statistical analysis was performed using a Mann-Whitney U test for non-parametric data. Measurements were made before any chest compressions.*

| Parameter | Fresh-Frozen Cadavers | Thiel Cadavers | *p* |
| --- | --- | --- | --- |
| PEEP total  (cmH_2_O) | 5.45 (4.85 - 5.93) | 1.85 (1.63 - 2.80) | < 0.001 |
| Plateau Pressure  (cmH_2_O) | 19.40 (17.47 - 20.00) | 15.05 (14.05 - 16.55) | 0.009 |
| Expiratory Tidal Volume  (mL) | 383.50 (361.25 - 420.00) | 293.50 (265.75 - 332.50) | < 0.001 |
| Airway Opening Pressure (AOP)  (cmH_2_O) | 2.45 (1.78 - 5.23) | 12.00 (8.75 - 13.63) | < 0.001 |
| Compliance without AOP  (mL/cmH_2_O) | 31.60 (27.86 - 34.38) | 22.50 (21.11 - 27.19) | 0.006 |
| Compliance with AOP  (mL/cmH_2_O) | 33.80 (30.78 - 35.72) | 68.64 (56.54 - 104.33) | < 0.001 |

**Appendix F.1 – Time variation of oxygen partial pressure (PaO2), carbon dioxine partial pressure (PaCO2) and end-tidal CO2 (EtCO2) between each sequence.**

*The first graph corresponds to the sequence with the flat position first and then head and thorax elevation position (HTE). The second graph corresponds to the sequence with the HTE position first and then the flat position.*

*Variables are presented as median (Q1 – Q3).*

**Appendix F.2 (bis) – Time variation of oxygen partial pressure (PaO2), carbon dioxine partial pressure (PaCO2) and end-tidal CO2 (EtCO2) between each sequence.**

*The first graph corresponds to the sequence with the flat position first and then head and thorax elevation position (HTE). The second graph corresponds to the sequence with the HTE position first and then the flat position.*

*Variables are presented as median (Q1 – Q3).*

**Appendix G – Pulmonary loss of lung volume stratified by cadaver.**

**Appendix H – ARRIVE Guidelines Checklist.**
